# A Targeted Mitigation Strategy for Freshwater Harmful Algal Blooms through the Release of a Small-Molecule Polyphenolic Algaecide from Clay Nanotubes

**DOI:** 10.1021/acs.est.6c03235

**Published:** 2026-07-16

**Authors:** Pedram AziziHariri, Borui Wang, Monica Brady, Istiak Hossain, Nicholas R. Sandoval, William Scott, Tim I. McLean, Vincent J. Lovko, Vijay T. John

**Affiliations:** † Department of Chemical and Biomolecular Engineering, 5783 Tulane University, New Orleans, Louisiana 70118, United States; ‡ Department of Biochemistry and Microbiology, North South University, Bashundhara, Dhaka 1229, Bangladesh; § Phytoplankton Ecology Research Program, Mote Marine Laboratory, Sarasota, Florida 33577, United States; ∥ Department of Medical Education, The University of Tennessee Health Science Center, Memphis, Tennessee 38163, United States

**Keywords:** halloysite nanotube, algaecide, harmful algal bloom, mitigation, Microcystis aeruginosa

## Abstract

Harmful algal blooms (HABs) caused by *Microcystis aeruginosa* (MC) pose serious threats to freshwater ecosystems, public health, and water treatment infrastructure. Here, we present a targeted mitigation technology involving flocculation and settling of MC using a flocculant, polyaluminum chloride (PAC), and a nanotubular clay, halloysite, which serves both as a ballast to aid settling of the flocs and as a reservoir to deliver algaecides. The tubular morphology of halloysite allows loading of algaecide, thus constituting a targeted release of algaecide to MC cells in the floc. The specific algaecide chosen is a small-molecule polyphenol, propyl gallate (PG), whose release can be modulated by coating the halloysite nanotubes (HNT) with a thin layer of paraffin wax. Pulse amplitude modulation fluorometry (PAM) measurements confirm that PG disrupts the photosynthesis efficiency of MC in doses as low as 25 mg/L. Importantly, the extensive extracellular polymeric substance (EPS) produced by MC forms a hydrated and adhesive matrix that physically entraps HNTs within the floc through entanglement and interfacial interactions, thereby enhancing localized algaecide delivery while limiting exposure to nontarget organisms. Our findings demonstrate the potential of HNT-based delivery systems as scalable, environmentally benign technologies for managing freshwater HABs caused by *Microcystis aeruginosa*, with promising implications for long-term bloom suppression and ecological safety.

## Graphical abstract

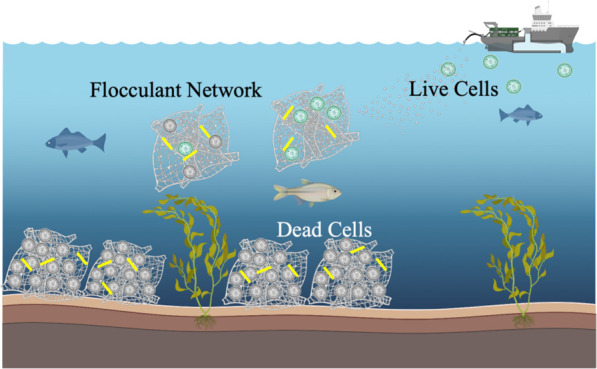

## 1 Introduction

In recent decades, the frequency and severity of harmful algal blooms (HABs) have increased worldwide^1,2^ due to climate change and eutrophication,^3,4^ making them a growing environmental and economic concern.^5,6^ HABs pose significant risks to public health and water quality through the production of toxins and taste- and odor-causing compounds, while also impacting fisheries, tourism, and water treatment systems.^7–12^ Among bloom-forming species, *Microcystis aeruginosa* is particularly problematic due to its production of intracellular toxins such as microcystin, as well as compounds that degrade water quality.^13^ Conventional mitigation strategies include both flocculation and settling,^14^ and chemical algaecidal treatment.^15^ While both treatments have been proven effective, the delivery of algaecide is often indiscriminately applied to the entire water column. This may result in the need for large algaecide concentrations and the potential to impact off- target organisms.

Managing HABs is therefore crucial for maintaining human and economic health in areas where they occur. Various chemical and biological mitigation strategies have been documented in the literature.^16–19^ Among these approaches, flocculation and sedimentation techniques stand out.^20–23^ Most commonly, these techniques utilize a cationic flocculant such as PAC combined with mineral clays to aggregate and sink the anionic cells.^20,24^ This process deprives the organisms of sunlight, disrupting their photosynthetic pathways and ultimately reducing their viability.^25–28^ Recent developments in these technologies involve incorporating an eco-friendly algaecide into the floc, enabling targeted elimination of harmful algal species.^29^

Here, we describe a new technology to treat harmful algal blooms (HABs) through the delivery of algaecides with low water solubility using a composite flocculation system. The low water solubility of an algaecide is a necessity for integration into a floc, as water-soluble algaecides including sodium percarbonate^15,30–32^ will dissolve into the water column too rapidly to be effective as a strategy to target cells trapped in a floc layer as it settles through the water column. Thus, by loading poorly soluble algaecides within the floc structure, this approach concentrates the active agent precisely to the captured algal cells and enables a sustained, diffusion-controlled release. As a result, the treatment becomes both more efficient and more environmentally selective, providing prolonged local exposure of algal cells to inhibitory algaecide concentrations while minimizing off-target distribution and unnecessary chemical loading of the ecosystem.

In this work, we consider a small molecule polyphenolic as the algaecide, focusing on propyl gallate (PG). Propyl gallate has a lower solubility in water (3.5 g/L) compared to other small polyphenolics such as gallic acid (12 g/L) and pyrogallol (400 g/L).^33,34^ These small-molecule polyphenols produce H_2_O_2_ in water through the mechanism shown in Supporting Information S1.^35,36^

H_2_O_2_ permeates the algal cell membrane and interacts with intracellular Fe^2+^, producing toxic hydroxyl radicals through the Fenton reaction (eq 1):

1 $${\mathrm{Fe}}^{2+}+H_{2}O_{2}\to {\mathrm{Fe}}^{3+}+\mathrm{HO}\cdot +{\mathrm{HO}}^{-}$$

with the hydroxyl radicals leading to DNA oxidation and cell death.^37–39^

In the technology described in this paper, PG is encapsulated in the lumen of a tubular nano clay, halloysite. Halloysite nanotubes (HNT) are natural clay minerals with the morphology of scrolled-up kaolinite sheets, leading to a lumen that is 20–40 nm in diameter.^40^ The length of HNT tubes varies from 0.5 to 2 μm, thus allowing HNT to sequester PG within and release it from the lumen. Additionally, our technology is based on a widely used flocculation and sinking technology involving the use of the cationic polyaluminum chloride (PAC) as a flocculant.^25,41–43^ Our hypothesis is that the silica-based HNT with anionic silanol surface groups will bind to PAC together with the cells, functioning as a ballast and enabling accelerated sinking of the floc.

The overall concept of this work is shown in Figure 1, where PG in the lumen of HNT is released into a floc containing trapped cells and acting as an algaecide.

**Figure 1:**
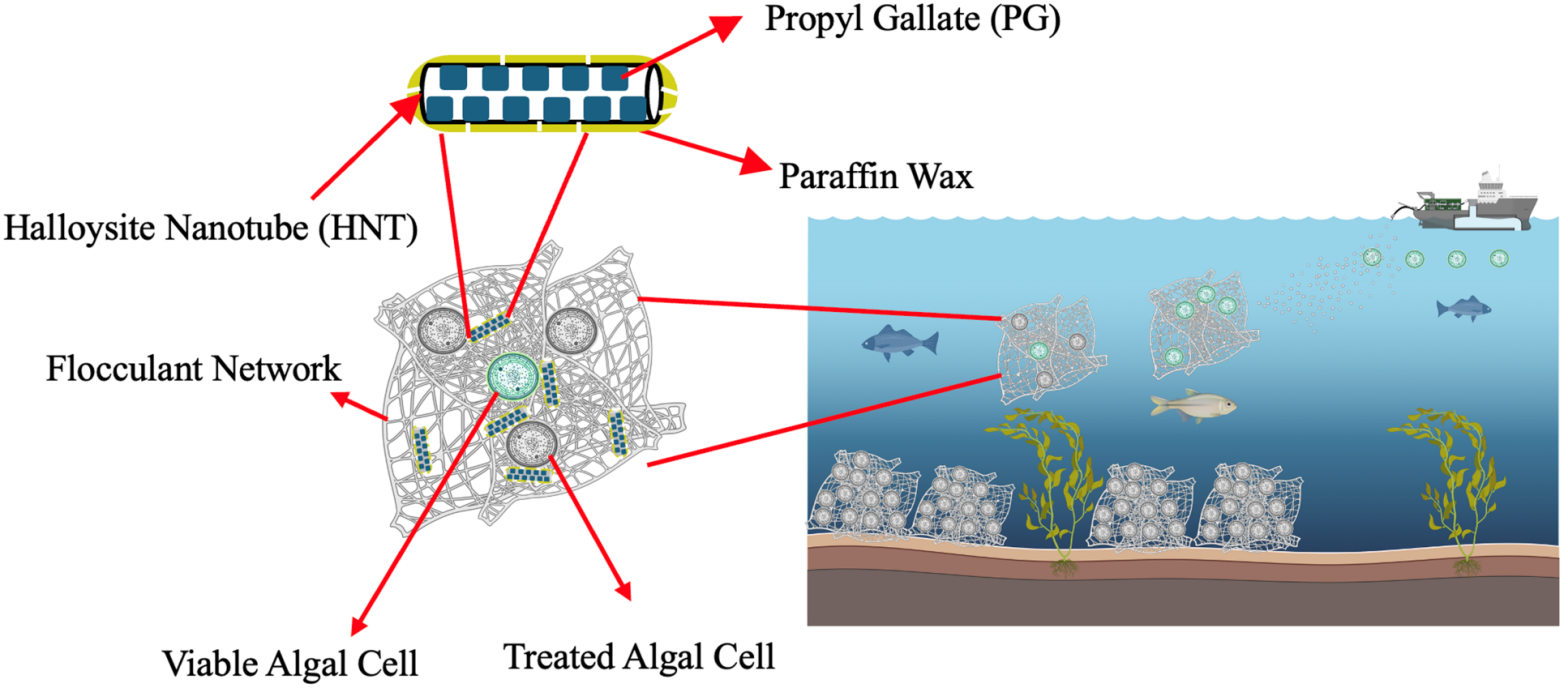
A schematic of algaecide delivery utilizing HNT. Viable cells are shown in green in the water column, and the dead cells are shown in the bottom in gray. The inset in the bottom left shows a schematic of the flocs containing HNT loaded with PG, and cells entrapped in them. The inset in the top left shows a more detailed schematic of HNT loaded with PG and coated with paraffin wax.

To control the release of PG from the lumen, we have further refined the technology through coating HNTs with a thin layer of wax^44^ that reduces lumen pore diameters and slows the release of PG. Such concepts of integrating particulate algaecides with flocculating agents ensure that the particulate algaecides are also trapped in the flocs, where cells will also be entrapped, leading to a new concept in the technology of flocculation and sinking of harmful algae, where the temporal release of hydrogen peroxide is localized to the flocs.^29,45^ By refining these techniques, we propose an effective and scalable strategy to mitigate the environmental and economic damages caused by HABs.

In this work, we aim to address the efficiency of mitigation by designing a system to enable rapid removal of algal cells from the water column via flocculation and sedimentation, and to suppress short-term algal regrowth through localized delivery of an algaecide within the floc matrix. The flocculation component targets immediate mitigation, while the controlled release of the algaecide is designed to inhibit residual cell viability and photosynthetic activity following sedimentation. We note that long-term bloom recurrence is beyond the scope of this study and is not directly evaluated here.

## 2 Materials and Methods

All chemicals were purchased from Sigma-Aldrich unless otherwise specified. PG and HNT were used as received. Sterile 50 mL conical tubes (Falcon, item no. 339653) were purchased from Thermo Scientific.

### 2.1 Cell Cultures and Treatments

An initial culture of *Microcystis aeruginosa* (strain LE3) was obtained from Mote Marine Laboratory, Sarasota, Florida, USA. MC cells were grown and maintained in BG11 media (sterile freshwater + Alga-Gro concentrated medium purchased from Carolina Biological Supply). Cell cultures were maintained under fluorescent lighting on a 16 h:8 h day:night regime (∼80–100 μmol photons m^–2^ s^–1^) at 25 °C in a diurnal incubator. Stock solutions of PG, PG loaded in HNT, and wax-coated HNT loaded with PG, where applicable, were freshly prepared using sterile freshwater purchased from Carolina Biological Supply. Four concentrations of PG (25, 50, 100, 200 mg/L) were selected for viability experiments. After the treatment, the centrifuge tubes were maintained in an incubator under a 16 h:8 h day:night cycle, and cell viability was assessed at fixed time points. Before withdrawing samples for cell enumeration, each treatment was gently mixed to resuspend the cells and to ensure a representative cell density within the sample. The effects of treatments on the cell density were investigated utilizing a flow cytometer. All viability experiments were conducted using 50 mL sterilized centrifuge tubes, each containing MC cells suspended in 30 mL of cell media with an initial cell abundance ranging from 7 × 10^5^ to 1 × 10^6^ cells/mL in the exponential growth phase.

### 2.2 Preparation of HNT-Based Formulations

Propyl gallate was initially dissolved in methanol. The concentration of propyl gallate in methanol used was 5 mg/mL during the loading process, and the loading ratio (PG:HNT by weight) was 0.5. PG encapsulation followed a simple procedure of dispersing HNT in a solution of PG dissolved in methanol, followed by evaporation of the methanol. Capillary forces drive the dissolved PG into the lumen, and we simply assume that PG is encapsulated in the lumen. For the wax-coated PG-HNT treatment, paraffin wax was dissolved in hexane and stirred for about 10 min. PG-HNT (0.5:1) composite was added to the solution and stirred for about 5 min. The flask was placed in a rotary evaporator operated at 40 °C to keep the wax completely solubilized in hexane. A vacuum was applied to the rotating system (rotating at 80 rpm) to allow the formation of wax coating on the HNT until all the hexane in the system was evaporated (200 mbar is sufficient for this procedure due to the high volatility of hexane). The samples were then kept in the vacuum oven overnight prior to use in further experiments.

HNT (or PG-HNT-wax) and PAC were premixed prior to addition to the algal suspensions. The mixing was done by dispersing the components in freshwater and vortex mixing for 30 s.

### 2.3 Optical Microscopy

All optical micrographs were obtained using a Nikon Eclipse TE2000-U inverted microscope. 4× and 10× objective lenses were used to image the flocs with the cells entrapped within them.

### 2.4 Viability Assessments

#### 2.4.1 Cell Counting via Flow Cytometry

The enumeration of cells was performed through cell counting by flow cytometry with an Attune NxT flow cytometer (Thermo Fisher). Initially, 500 μL of samples were collected and centrifuged at 14,000 RCF, followed by removal of the supernatant. Next, 1 mL of freshwater (Spring water purchased from Carolina Biological Supply) was added to the pellets, and the pellets were resuspended. 100 μL of the suspension was withdrawn and mixed with 900 μL of 1X PBS buffer. The mixture was inserted into a 5 mL sterile culture tube. A blue solid-state laser (488 nm excitation) was used to excite the cells. 50 μL of each sample was taken up for analysis. Using a population of cells growing in the exponential phase, we defined the population gate by gating 100% of exponentially growing MC cells on the FSC vs SSC cytograms. Cell enumerations were performed under a flow rate of 500 μL/min on the defined gates.

#### 2.4.2 Chlorophyll-*a* Concentration

Chlorophyll-*a* (Chl-*a*) was extracted using an 80% acetone solution.^46^ Briefly, 2 mL of cell suspension was collected and transferred to 2 mL centrifuge tubes, and centrifuged at 14,000 RCF for 5 min at room temperature, followed by removal of the supernatant. 0.5 mL DI water was added to each tube, and the pellets were resuspended, followed by heat-treating the cell suspensions in boiling water for 3 min. The suspensions were then allowed to cool down, and 2 mL of cold acetone was added to each of the suspensions, followed by incubation in the dark for 30 min in glass vials. Lastly, samples were transferred to centrifuge tubes and centrifuged at 14,000 RCF for 5 min before running UV–vis. The baseline was calibrated against 80% acetone solution in DI water. The UV–vis spectra were recorded (by a Shimadzu UV-1700 spectrophotometer), and absorbance values at 663 and 645 nm were measured. The chl-*a* levels (μg/L) were calculated using the following equation (eq 2):^46^

2 $$\left[Chl‐a\right]=12.7\;\lambda_{663}‐2.69\;\lambda_{645}$$

#### 2.4.3 Pulse-Amplitude-Modulated (PAM) Fluorometry

To determine the effect of PG on MC photosynthetic performance, PAM fluorometry was used to measure the maximum quantum efficiency (F_v_/F_m_) of photosystem II in the cells exposed to different concentrations of PG. Samples were dark-adapted for approximately 20 min before measurements were taken. The value of F_v_/F_m_ was recorded for each dark-acclimated sample after exposure to a saturating light pulse.^47^ All treatments were run in triplicate, with averages and standard deviations calculated. Analyses were conducted at Mote Marine Laboratory using a PHYTO-PAM-II (compact version) fluorometer (Walz, Germany).

### 2.5 Transmission Electron Microscopy (TEM)

The morphologies of pure HNT, HNT loaded with PG (0.5 PG:1 HNT wt), and wax-coated PG-HNT composites were characterized by using a FEI G2 F30 Tecnai transmission electron microscope operated at 300 kV to verify the presence of PG within HNT lumens. Samples were initially dispersed in hexane (except for the wax-coated composite, which was dispersed in water) due to the insolubility of PG in hexane. Five μL of each sample was then pipetted onto the surface of a 200-mesh lacey carbon-coated copper grid (from Ted Pella, Inc.). Excess hexane was then evaporated, and the dried specimen was transferred onto a single-tilt specimen holder for imaging.

### 2.6 Scanning Electron Microscopy (SEM)

To characterize the morphologies of pure HNT and HNT loaded with PG (0.5 PG:1 HNT wt) and to verify the absence of PG large deposits outside of the HNT lumens and successful encapsulation of PG within the HNT lumens, a field emission scanning electron microscope (Hitachi S-4800) operated at 3 kV was used. Samples were coated with graphite before insertion into the sample chamber to eliminate any charge buildup during imaging.

### 2.7 UV–Visible Spectroscopy

The release of PG from loaded HNT with and without the paraffin wax coating was quantified with a UV–vis spectrophotometer (Shimadzu UV-1700). PG has a characteristic peak in the UV–vis spectrum around 275 nm. The UV–vis spectrum and the calibration curve can be found in the Supporting Information (S2). The molar extinction coefficient was measured ε = 9.76 mM^–1^·cm^–1^ at λ = 275 nm based on the calibration curve. In short, samples were collected at different time points, and the absorbance was measured at λ = 275 nm. The media used in this experiment was the freshwater (Spring water purchased from Carolina Biological Supply) used for cell cultures. The concentration of PG was then calculated based on the calibration curve obtained through earlier experiments.

### 2.8 Statistical Analysis

All experiments were conducted in triplicate (n = 3), and that error bars shown in the figures represent standard deviation. The replicates correspond to biological replicates (separate cultures). Statistical analyses were conducted using GraphPad (Prism 10). One-way analysis of variance (ANOVA) with multiple comparisons was employed to compare statistically significant differences between control and treatment groups at each time point. Significance thresholds were set at *p* < 0.05 for statistically significant (*), *p* < 0.01 for highly significant (**), *p* < 0.001 for very highly significant (***), and *p* < 0.0001 for extremely significant (****) results.

## 3 Results and Discussion

### 3.1 Impact of Propyl Gallate (PG) on Cell Growth

The effects of PG were assessed against the freshwater cyanobacteria *Microcystis aeruginosa* to investigate its algaecidal activity. Four concentrations of PG (25, 50, 100, 200 mg/L) were tested over 168 h, and the density of the cells was assessed through flow cytometry. The cell density as obtained through flow cytometry is reported in Figure 2a. Throughout the experiment, the control group shows a steady, significant rise (∼1900% in 168 h) in cell density compared to the initial cell density (1 × 10^6^ cells/mL shown by the dotted horizontal line) in Figure 2a, indicating optimal conditions for cell growth. A minimum concentration of 25 mg/L is shown to inhibit the growth of MC, and the higher concentrations show a similar impact on growth suppression throughout the 168 h period. The effect of lower concentrations was also evaluated and is shown in the Supporting Information S3, with, concentrations below 25 mg/L showing no growth suppression. Figure 2b illustrates the overall Chl-*a* production by MC in the control and the treated groups. The control group shows a consistent increase (∼1400% in 168 h) in Chl-*a* as the cell count in the culture increases, while a concentration of 25 mg/L of PG halts the production of Chl-*a* in MC, with no sign of recovery after a week. Higher concentrations of PG show a similar impact on Chl-*a* production, simply halting the production of Chl-*a*.

**Figure 2:**
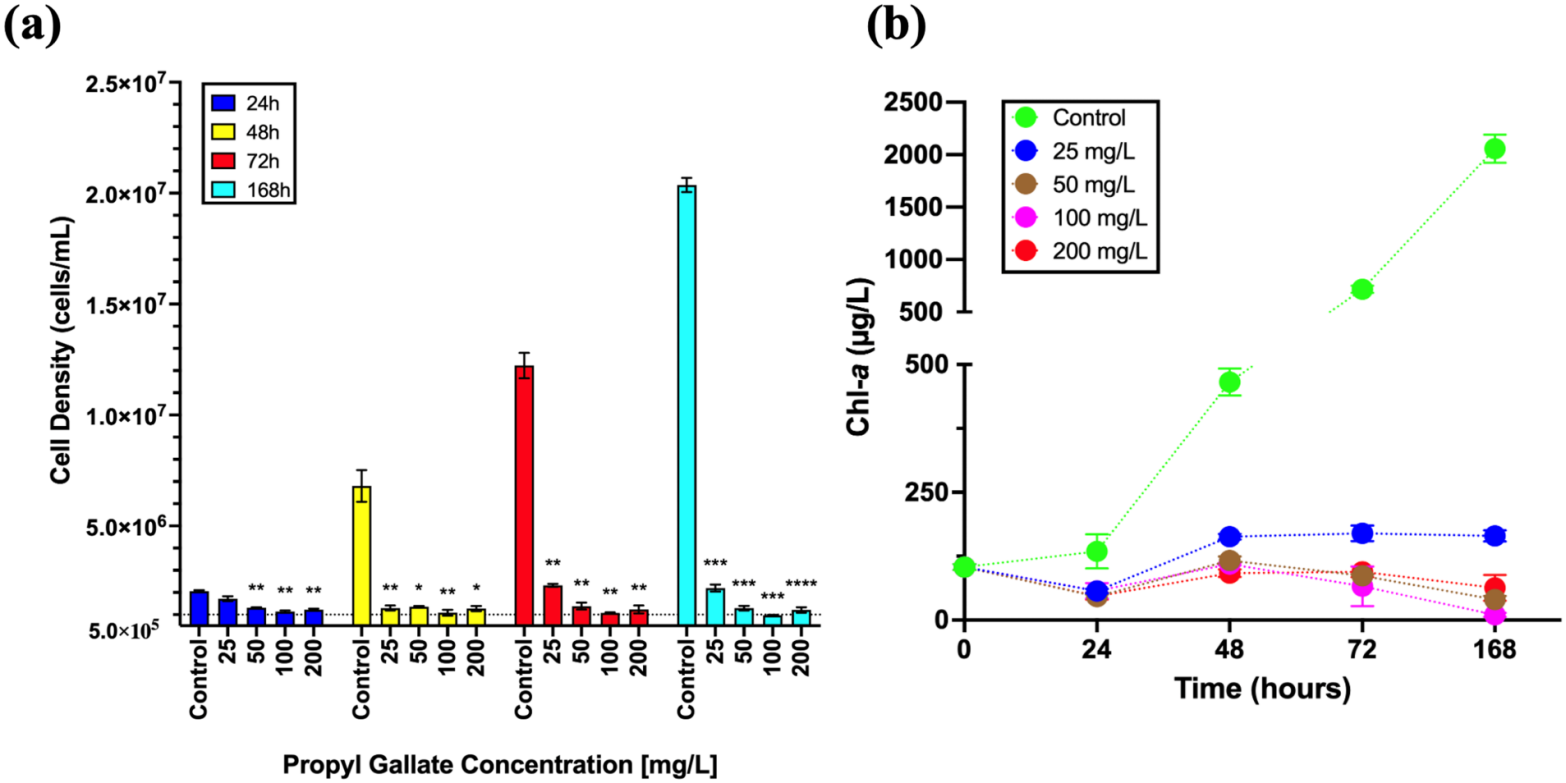
(a) Effect of propyl gallate on the cell density of MC at different concentrations (25, 50, 100, 200 mg/L). Concentrations as low as 25 mg/L are shown to inhibit the growth of cells, when higher concentrations show more significant growth inhibition within the first 7 days; (b) extracted Chl-*a* from MC shows that a concentration as low as 25 mg/L PG slows down the production of Chl-*a* throughout the seven-day period, while the control group shows a sharp increase in production of Chl-*a*.

### 3.2 Pulse-Amplitude-Modulated (PAM) Fluorometry

To better understand the inhibition of cell growth without destruction of the cells, we conducted a separate set of experiments at the same PG concentrations (25, 50, 100, 200 mg/L) using PAM to characterize photosystem II (PS II) efficiency.^47^ Figure 3a illustrates the PAM results as characterized by F_v_/F_m_, the maximum quantum yield efficiency of PS II. MC cells in the control samples exhibited significant photophysiological activity, as demonstrated by their F_v_/F_m_ values. As shown in Figure 3a, PG significantly reduced F_v_/F_m_ values (∼33% at 50 mg/L) within 3 h of PG introduction, indicating severe inhibition of PS II verifying the efficiency of PG to impair photosynthesis almost 100% in concentrations as low as 25 mg/L. No apparent recovery was observed within 48 h of the experiment. The readings from fluorometry, shown in Figure 3b, agree with our findings from extracting Chl-*a* and quantifying the concentration based on its absorbance. The fluorescence in the control sample followed an increasing trend over 144 h (∼270%), yet the treatments show no increase in fluorescence from chlorophyll, as the Chl-*a* production is affected by the PG treatments. These experiments indicate that PG is effective as a significant inhibitory compound to the growth of MC and dramatically suppresses photosynthetic efficiency.

**Figure 3:**
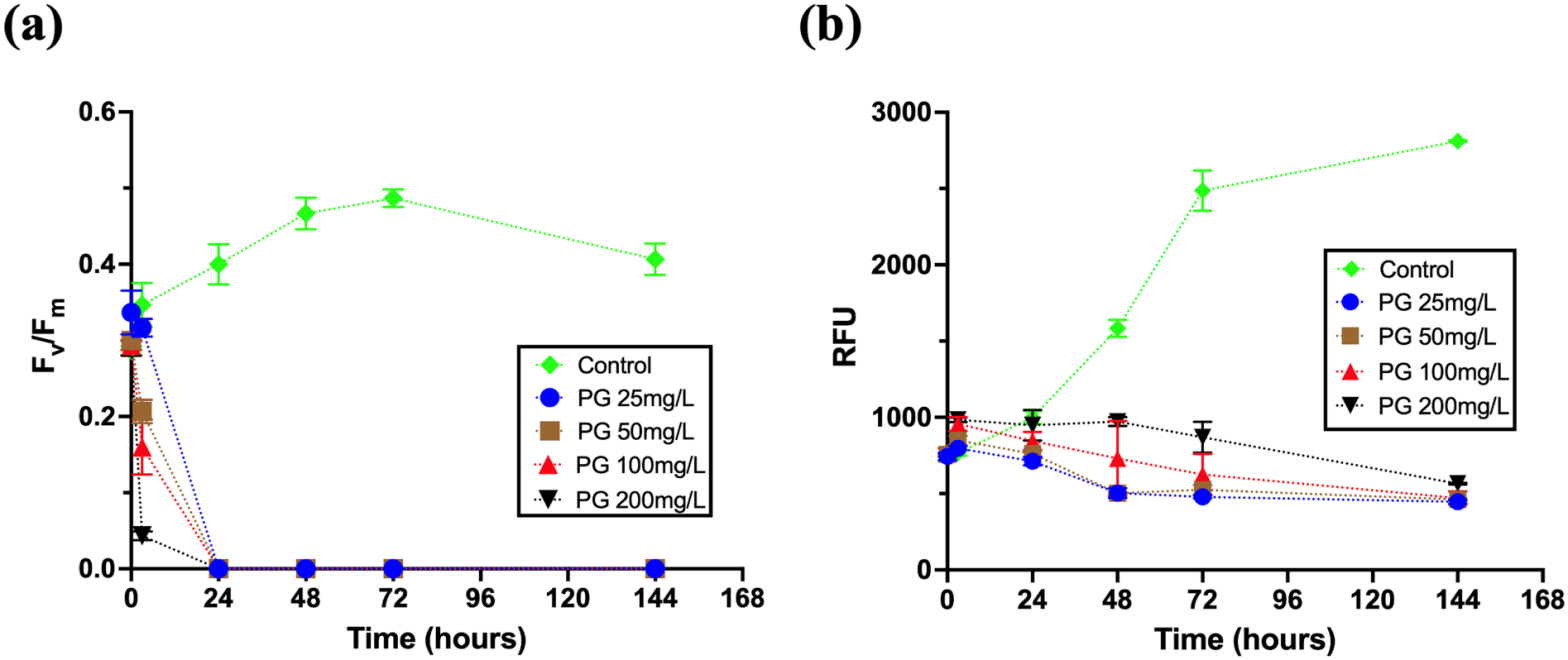
(a) PAM readings of MC treated with various concentrations of PG ranging from 25 mg/L up to 200 mg/L. A concentration of 50 mg/L is effective in only 3 h to cut the PSII efficiency 33%. Concentrations as low as 25 mg/L are found to completely reduce the PSII efficiency within 24 h; (b) The relative fluorescence of the samples used for PAM over the same period. Similar to Chl-*a* levels in Figure 2b, RFU levels increase in the control group, whereas the treated samples slightly lose their fluorescence.

The next step is to develop a targeted delivery of PG to MC, which requires having the cells entrapped in a network of a flocculant, where the algaecide is applied directly to the cells, with minimized impact on nontarget organisms. Hence, we evaluated the flocculating efficiency of PAC, and PAC combined with HNT. HNT acts as a ballast and accelerates the settling of the PAC flocs as they are formed in the water.

### 3.3 MC Flocculation with PAC and HNT

Halloysite is an aluminosilicate similar to kaolinite, but it consists of sheets that scroll up to a tubular structure with an inner lumen, forming the nanotubes.^48^ Scanning and transmission electron micrographs of HNT are illustrated in the Supporting Information S4. PAC is a highly cationic compound with the chemical formula: [Al_2_(OH)_*n*_Cl_6–n_]_m_ (1 ≤ *n* ≤ 5, *m* ≤ 10). It is widely used as a flocculant in water treatment due to its low cost^41^ and has been utilized in numerous HAB mitigation technologies due to its ability to electrostatically bind to the anionic algal cells.^25,41^ In more common flocculation and sinking technologies, surface silanol-containing anionic clays such as kaolinite are typically added to PAC^21,43^ and electrostatically attach to the PAC, acting as ballast to accelerate the sinking process. The external surface of HNT is also anionic, with a negative zeta potential of approximately −30 mV at pH = 8.^49^ We expect HNT to similarly become entrapped in a PAC floc and have therefore investigated the flocculation and sinking of MC with PAC and HNT. With a solid density of HNT of 2.6 g/mL, the incorporation of HNT in the floc serves as a ballast to enhance settling.

Figure 4 a shows an optical micrograph of PAC flocs with HNT, where MC is captured in the flocculant network. The presence of cells within the floc is evidenced in this micrograph, where cells are easily detected as green dots (shown by the arrows). The inset shows the cells entrapped in PAC + HNT flocs and settled at the bottom of a 50 mL centrifuge tube, where the green color indicates the presence of MC cells within the settled floc. To verify how much PAC is required for successful flocculation of MC, a concentration-dependent experiment was performed, the results of which are illustrated in Figure 4b. The HNT concentration was kept constant at 100 mg/L, and the PAC concentration was varied from 50 mg/L to 100 mg/L. At specific time points, samples were collected from the supernatant, and cell enumeration was done through flow cytometry. The combination of 100 mg/L PAC and 100 mg/L HNT is effective in removing essentially 100% of the cells in 3 h, and there is no cell escape from the flocs in 24 h.

**Figure 4:**
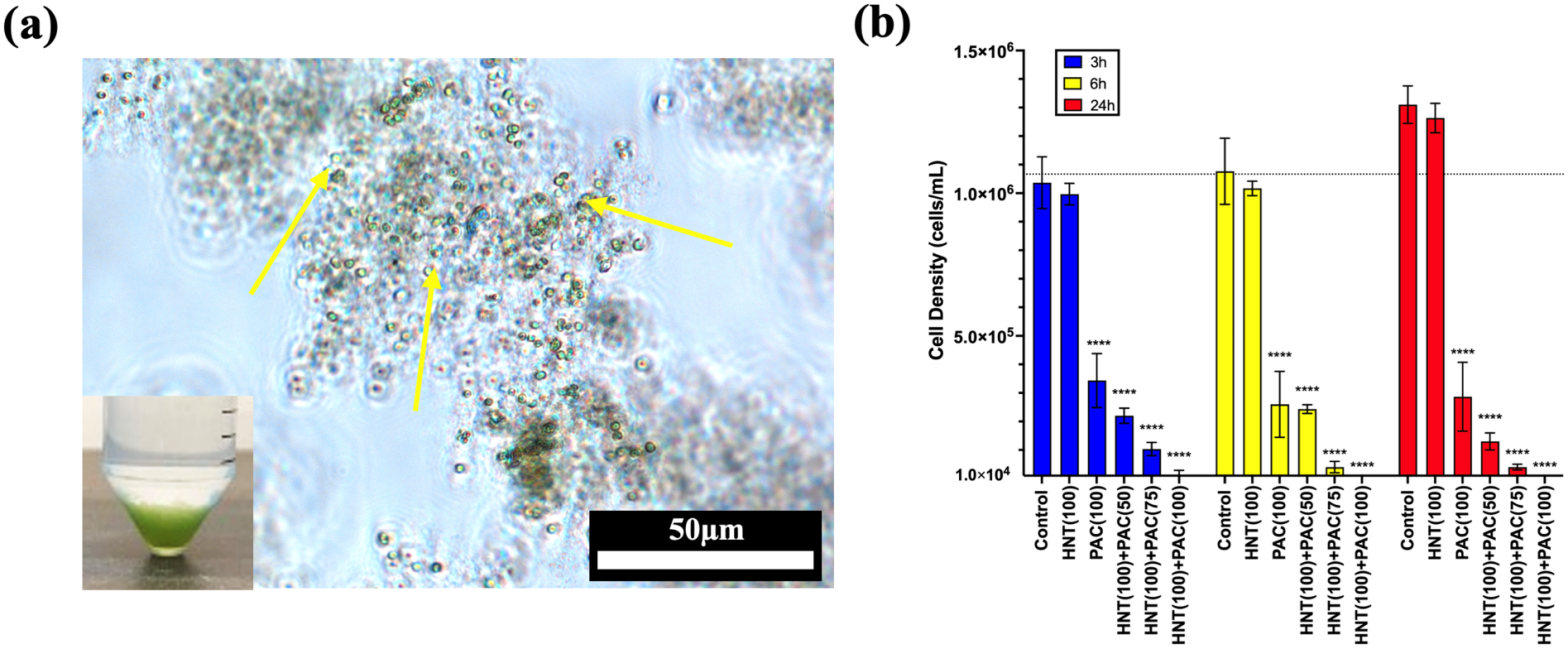
(a) An optical micrograph of cells entrapped in 100 mg/L PAC flocs where 100 mg/L HNT was added as a ballast (the inset is an optical image of the same floc settled at the bottom of a 50 mL centrifuge tube, where the MC presence is evident by the green color of the settled floc); (b) the effect of adding PAC and HNT on cell density. All concentrations (in parentheses) are in mg/L. Almost 100% cell removal is achieved within 3 h at HNT = 100 mg/L and PAC = 100 mg/L.

### 3.4 Encapsulation of PG in HNT and Delivery to Cells in a Floc

Loading of PG in the lumen of HNT using PG dissolved in methanol is feasible due to the high capillary pressures driving solvent into the nanotubes. An approximate capillary pressure can be calculated using the Young–Laplace equation:

3 $$P_{c}=\frac{2\gamma \cos\theta }{r}$$

where:

P_c_: capillary pressure (Pa),

γ: surface tension of the solvent (N/m)

θ: contact angle of the liquid with the nanotube wall, assumed to be 0 for complete wetting

*r*: radius of the nanotube (m)

Assuming complete wetting with a methanol surface tension of 0.022 N/m, and a nanotube radius of 10 nm, the equation yields a maximum value of 4.36 × 10^6^ Pa (43 atm) for the capillary pressure, which is the driving force for solvent intrusion into the lumen. Thus, the process of allowing PG dissolved in methanol to contact HNT, followed by slow solvent evaporation and drying, should lead to the loading of PG in the nanotube. Figure 5a shows an SEM micrograph of HNT loaded with PG. The lack of surface agglomerates of PG perhaps indicates that PG has entered the lumens or formed a thin coating on the external surface of HNT. Figure 5b is a direct TEM of the nanotubes showing a darkening of the lumen region, indicating ingress and encapsulation of PG. The thin film (red arrows) seen on the surface of a nanotube may represent PG on the external surface. In this work, we have used a loading ratio (wt ratio of PG to HNT) of 0.5 to minimize external accumulation of PG and the formation of external PG aggregates. Upon increasing the loading ratio, deposits of PG appear on the outer surfaces of the nanotubes (Supporting Information S5).

**Figure 5:**
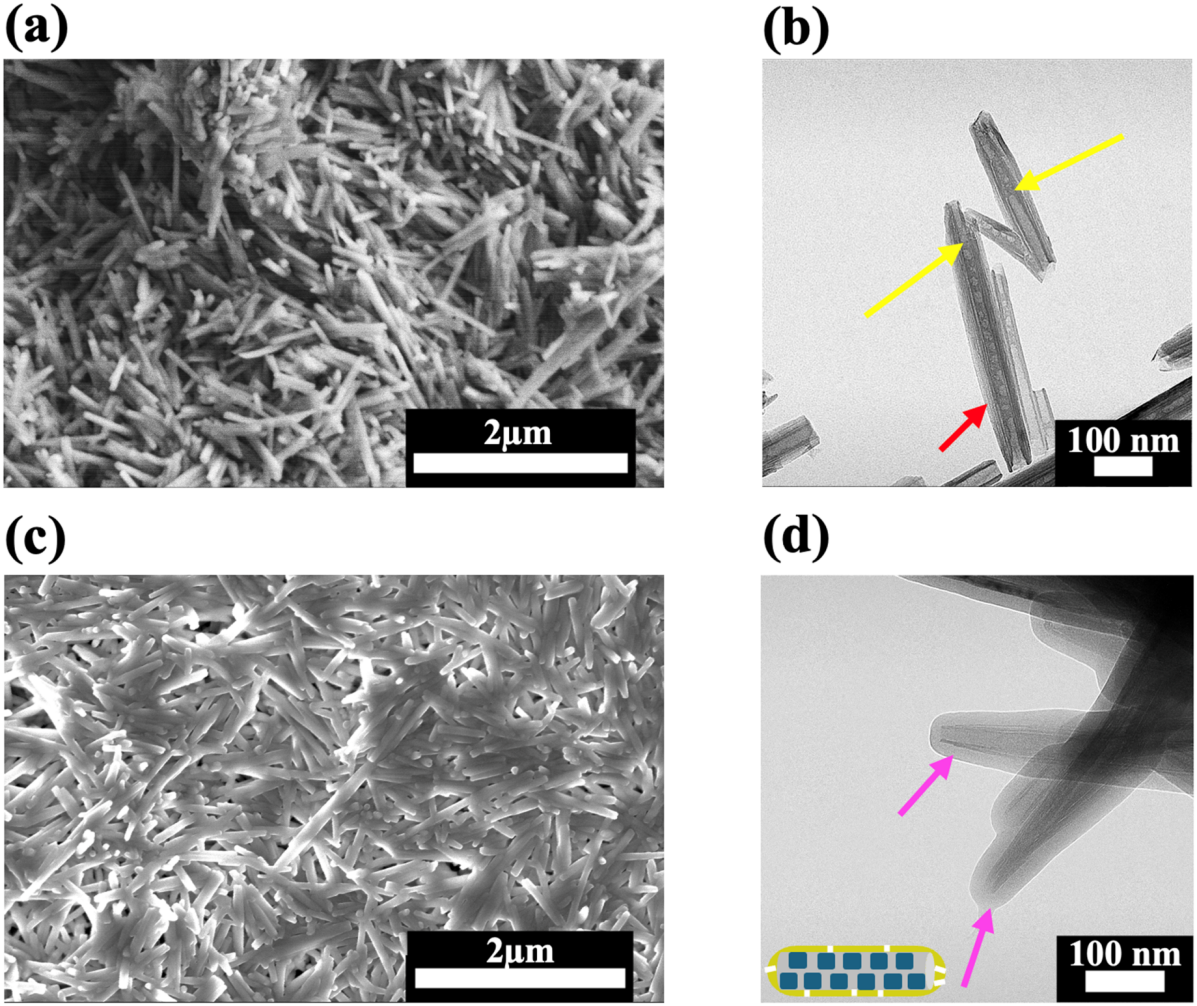
Electron micrographs of PG-HNT composite: (a) an SEM image of HNT loaded with PG. The absence of large PG deposits outside the lumens verifies the successful encapsulation of PG within the lumens; (b) a TEM image of PG-HNT composite, where the lumens are filled with PG, evidenced by the contrast (shown with yellow arrows), while the PG deposited outside of the lumens is shown with red arrows; (c) an SEM micrograph of HNT coated with paraffin wax; (d) a TEM of HNT loaded with PG and coated with paraffin wax. The magenta arrows show the uniform paraffin wax coating applied to the nanotubes to slow down the release of PG. A schematic of the wax-coated PG-HNT composites is also shown.

Figure 5c and 5d illustrate the design of wax-coated PG-HNT composites, and we defer the discussion of these figures after considering the release characteristics of PG as shown in Figure 6. In the wax-free system shown by the red curve in Figure 6, we see that there is a rapid-burst release of PG, indicative of the finite solubility of PG in water (3.5 g/L).^33,50^ We assume that within the confined volume of the nanotube, the dissolution rate is governed by the mass transfer across a stagnant layer where the concentration gradient is relatively low and the saturation solubility reached at the algaecide particle-fluid interface. The wax coating reduces diffusion rates at the nanotube lumen entrance/exit and may thus lead to a slower transport from the reservoir of solid PG trapped in HNT.

**Figure 6:**
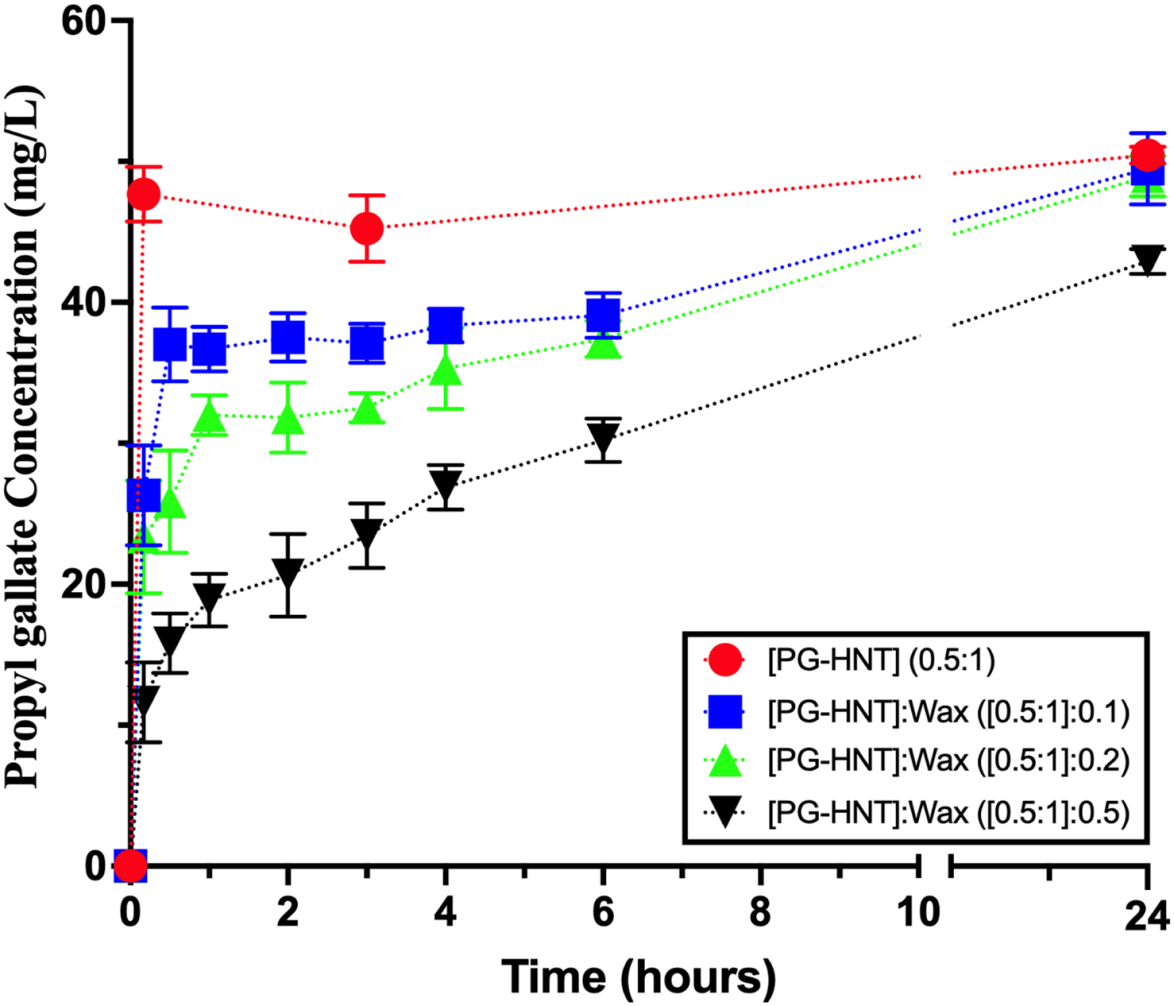
Release profile of PG from HNT loaded with PG. In the absence of wax, PG is released almost immediately from the composite. The wax coating slows down the release rate of PG. The final concentration of PG after it is completely released from the composites in this experiment is 50 mg/L.

To slow down the release of PG, we have coated the HNT with wax following a procedure where the HNT loaded with PG is immersed in paraffin wax dissolved in hexane and then removed, allowing the hexane to evaporate and the wax to form a coating on the HNT (Materials and Methods section). Figure 5c is the SEM image of the wax-coated PG-HNT composites, where the overall morphology of the HNT is preserved with no significant morphological distortions due to surface aggregation of wax. Figure 5d is a transmission electron micrograph of the paraffin wax coating surrounding the nanotubes. We note the capping of the ends and a coating around the nanotubes, which may slow the release of PG both from the lumen and from PG adsorbed to the external surface of the nanotubes.

Figure 6 illustrates the release characteristics of PG from HNTs with various levels of wax coating. The release of PG was quantified in freshwater (Spring water used for cell cultures purchased from Carolina Biological Supply). The final concentration of PG was designed as 50 mg/L after complete release. PG was loaded into HNT at a mass ratio 0.5, and the amount of each composite (with or without a wax coating) was adjusted so that the final concentration of PG after complete release would be 50 mg/L. As noted, in the absence of a wax coating, there is a rapid burst release whose magnitude is significantly delayed with increasing amounts of the wax coating. With a composite:wax ([PG-HNT]:wax) ratio of ([0.5:1]:0.5), the sustained delivery of PG takes place over a 24 h period, with only ∼50% of the PG content released within the first 3 h. After 24 h, the composites coated with the higher wax content, [PG-HNT]:Wax ([0.5:1]:0.5), release 80% of the PG content, while the other groups release the total designed PG content of 50 mg/L. This delay allows the operation of a process where the wax-coated HNT containing PG is mixed with PAC and delivered to the bloom. The PG release from HNT in the floc then becomes targeted to the cells in the floc. We thus note the relevance to developing a coating to delay release. In the absence of a coating, the PG is released in the precursor solution and loses its relevance to a targeted release to cells in a floc.

Figure 7a shows the overall MC cell density over a seven-day nonflocculation study to characterize the effect of PG release from HNT. In addition to the growth of the MC culture, an additional control of HNT and wax with no PG loading was included. As shown in Figure 7a, both of the control groups indicate robust growth. The inclusion of PG in the HNT suppressed MC growth, which was maintained over the course of the experiment. Figure 7b shows a continuously increasing level of Chl-*a* in the control groups but a complete suppression of additional Chl-*a* in the cultures subjected to PG released from HNT.

**Figure 7:**
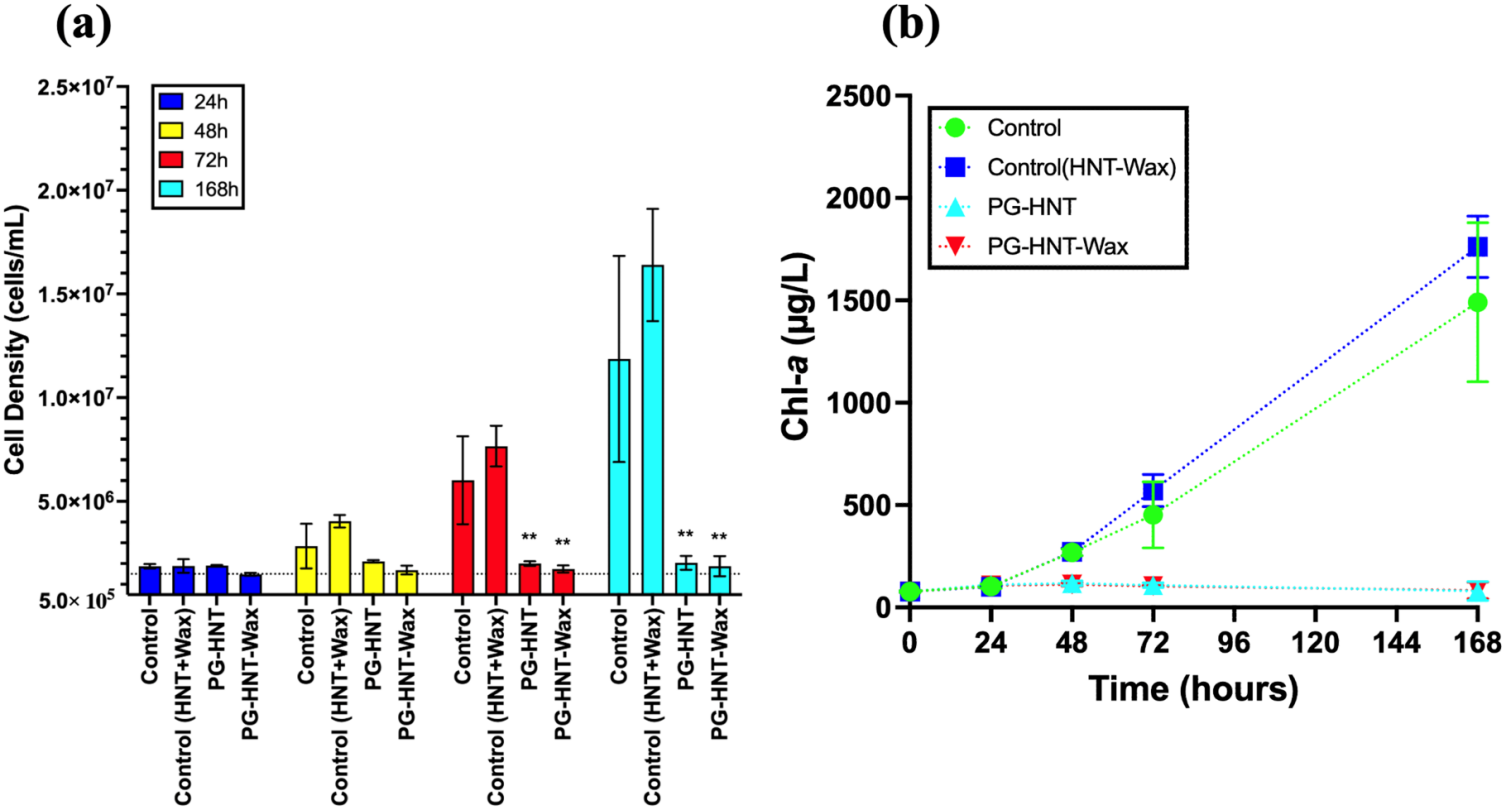
(a) Impact of HNT (100 mg/L across all samples) loaded with propyl gallate (50 mg/L) on the growth characteristics of MC; (b) Chl-a levels show a continuous growth in the control group, while in the treated groups, Chl-a production is inhibited.

We thus have the observation of flocculation and sinking of MC in PAC + HNT systems and the effective delivery of PG to MC through the use of wax-coated HNT to severely inhibit growth and cell photosynthetic efficiency. The overall coupling of PAC + HNT flocculation and sinking with the algaecidal activity of PG release from wax-coated HNT is shown through the images of Figure 8. Thus, the SEM of Figure 8 a illustrates wax-coated HNT decorating MC cells that are covered by PAC. We note that the cells can be clearly identified, but the surface morphologies are difficult to visualize since they are covered with the PAC mesh. The inset in the bottom left is an SEM image with higher resolution to show further details of HNT entrapped with the cells, and the inset in the top right is the optical micrograph of the flocculated cells to indicate the outlines of the floc. Figure 8b is a transmission electron micrograph of a cell entrapped in a PAC network where the wax-coated PG-HNT composites are also present. Figure 8c illustrates cell counts in the supernatant following treatment with PAC + PG-HNT-wax. As expected, the results confirm near-complete removal of cells from the water column due to flocculation, consistent with Figure 4 b. Figure 8c demonstrates that the PAC + PG-HNT-wax system effectively removes Microcystis cells from the water column while maintaining low cell densities over the seven-day observation period. We note that the PAC + HNT experiments presented in Figure 4 were designed to evaluate flocculation and settling efficiency and therefore focused on the initial 24 h, consistent with the primary objective of quantifying rapid cell removal. Specifically, the independent experiments in Figures 2, 3, and 7 demonstrate that PG, whether added directly or released from HNT, induces sustained inhibition of cell growth, Chl*-a* production, and photosystem II activity. Together with the flocculation results, these findings support the conclusion that the PAC + PG-HNT-wax system provides two complementary functions: rapid removal of cells through flocculation and sedimentation, followed by localized delivery of PG within the settled floc to suppress short-term regrowth. While flocculation-induced factors such as reduced light availability, confinement, and oxygen limitation may also contribute to postsettling stress, distinguishing their relative contributions from those of PG will require future studies incorporating an extended PAC + HNT flocculation control.

**Figure 8:**
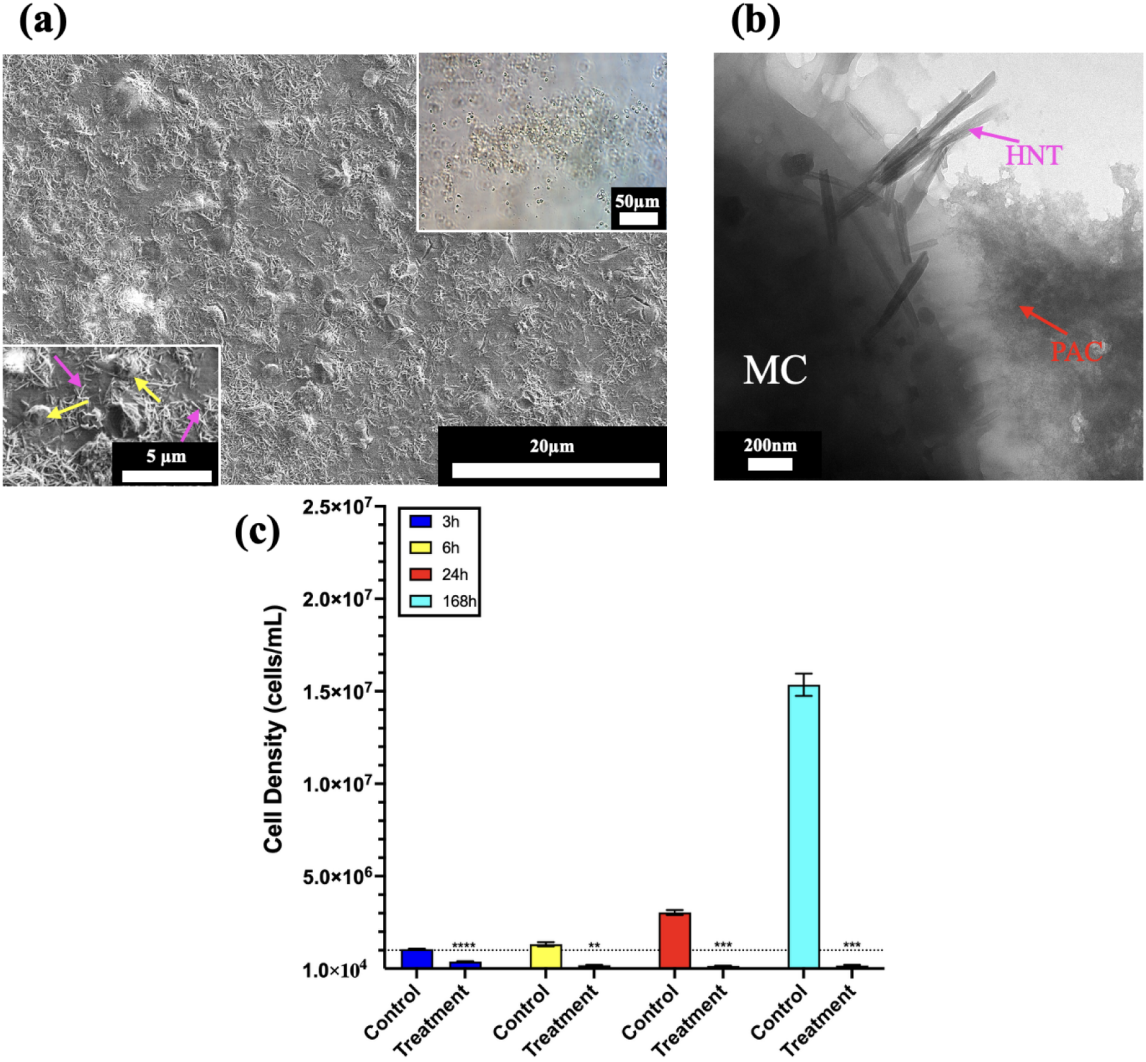
(a) SEM image of MC cells treated with wax-coated composites loaded in PAC flocs. The inset in the bottom left is at higher resolution where the magenta arrows show the wax-coated PG-HNT and the yellow arrows show the cells entrapped in the PAC network. The inset in the top right is an optical micrograph of the same sample, where cells are observed as tiny green dots clustered in a floc; (b) TEM image of MC cells treated with wax-coated HNT loaded in PAC flocs. The PAC network is shown with the red arrow, and the HNTs with a magenta arrow, with HNT entrapment by the EPS visualized; (c) the effect of PAC [100 mg/L] with (PG-HNT)-Wax [225 mg/L] treatments on the cell density in the water column as a function of time. The treatment suppresses cell resurgence for a week.

The presence of extracellular polymeric substances (EPS) is apparent in the micrographs, and some HNT appear to be localized within this matrix. EPS, primarily composed of polysaccharides, proteins, lipids, and nucleic acids, forms a hydrated and adhesive three-dimensional network that can entrap particulates.^51^ While the images suggest that EPS may contribute to the retention or association of HNT within the floc structure, these observations are qualitative in nature and do not provide quantitative measurements of HNT entrapment. Additional studies, such as spectroscopic characterization, surface charge analysis, or targeted binding assays, would be required to quantitatively elucidate EPS–HNT interactions and confirm their role in the system.

The use of PAC as a flocculating agent with HNT as a ballast removes over 98% of the cells with initial concentration of 1 × 10^6^ cells/mL. PG loading levels of 50 mg/L appear to be sufficient to cause a full loss of PSII efficiency in 24 h with the loss of efficiency observed for a week. The results therefore indicate that release of PG through HNT in a floc will be sufficient to create localized concentrations of PG sufficient to sustain a loss of cell viability over sustained periods.

In the environment of the floc, it is possible that the localized release of PG will lead to PG binding to the cell EPS layers through hydrogen bonding. The release of H_2_O_2_ may allow rapid diffusion through the cell wall and destruction of the cell. Quinones that are formed through PG oxidation may also bind to the EPS constituents. It is therefore important to measure both PG and quinone levels in the water column as a function of time to understand escape of these components from the floc. While quinones can be toxic to aquatic organisms,^52–55^ they are strong Michael acceptors and rapidly react with environmental nucleophiles, including dissolved organic matter (DOM) thus reducing their potential toxicity.^51,56,57^ The release of PG from HNT and the localization of PG release to the floc implies that the electrophilic quinones are subject to nucleophilic reaction with the EPS layers of MC cells that are concentrated in the floc. Understanding the fate of the polyphenol and the resulting quinones in the vicinity of cells of MC would be extremely worthwhile in translation of the concept to practice.

Traditional HAB mitigation approaches, including flocculation using PAC or clay minerals alone, are effective for the rapid removal and sedimentation of cyanobacterial cells, but do not address the destruction of the cells, some of which may be able to break free from loose flocs.^58^ Indiscriminate algaecide delivery could impact off-target organisms and lead to excess concentrations being applied.^59–61^ The targeted release of algaecide directly to cells in a floc could be helpful in sustained mitigation. However, there are limitations, since cells not captured in the flocs could reestablish blooms. It may be necessary to carry out repeated treatments to fully suppress the potential for bloom resurgence.

We note that hydrogen peroxide generation is strongly pH dependent. As the reaction mechanism in Supporting Information S1 indicates, the main reactive species is the dianion formed by deprotonation of the phenolic groups. In acidic media (pH < 6), the dominant species is neutral PG which leads to negligible H_2_O_2_ formation. In neutral to slightly basic pH (7–8), the dominant species is the monoanion which leads to slow H_2_O_2_ formation. At higher pH values (>9) the dianion dominates and H_2_O_2_ formation becomes rapid. Our systems are at pH values of 7–7.5 and we expect the formation of H_2_O_2_ to be very slow. Furthermore, the localization of PG oxidation within the floc may result in direct H_2_O_2_ contact with the cells and thus impact cell viability.

Cell concentrations on the order of 10^6^ cells mL^–1^ are representative of severe *Microcystis aeruginosa* bloom conditions reported in eutrophic waters^62,63^ and are associated with significant ecological and public health impacts, including cyanotoxin production, deterioration of water quality, and disruption of aquatic ecosystems.^64–66^ Although the present system did not produce a substantial decrease in measured cell density, PAM fluorometry demonstrated a pronounced loss of photosynthetic activity, indicating loss of cellular viability. Because Microcystis possesses a relatively robust cell wall structure, immediate cell lysis is not necessarily expected under these treatment conditions. Therefore, maintenance of cell counts should not be interpreted as persistence of active or viable cells, but rather as the presence of structurally intact yet physiologically inactive biomass.

The results presented here demonstrate a combined mitigation strategy that integrates rapid algal removal with suppression of post-treatment regrowth. Specifically, PAC + HNT enables efficient flocculation and sedimentation of *Microcystis aeruginosa*, addressing the need for immediate bloom mitigation. Concurrently, the controlled release of PG from wax-coated HNT within the floc significantly inhibits photosynthetic activity and cell growth, reducing the likelihood of short-term regrowth from sedimented cells. While this study demonstrates effective short-term suppression of algal growth following flocculation, long-term bloom recurrence under environmental conditions and optimization of HNT dosage represents an important direction for future work, particularly for balancing material usage, delivery efficiency, and cost in field-scale applications.

This work demonstrates a proof-of-concept strategy for coupling flocculation with controlled algaecide delivery for the mitigation of *Microcystis aeruginosa*. Halloysite nanotubes loaded with propyl gallate and incorporated into PAC flocs enabled effective removal of algal cells from the water column while also suppressing algal growth and photosystem II activity. The paraffin wax coating on PG-loaded HNT provided sustained release behavior, supporting prolonged exposure of *Microcystis aeruginosa* cells to inhibitory concentrations of propyl gallate over time. Microscopy observations suggest that extracellular polymeric substances (EPS) produced by Microcystis may contribute to entrapment of HNT within the extracellular matrix, potentially promoting close association between the delivery system and algal cells.

These findings demonstrate the feasibility of using naturally occurring halloysite nanotubes as carriers for localized algaecide delivery within flocculated biomass and support the potential of integrating HNT-based composites with flocculation-based HAB mitigation approaches. Continuing studies indicate the need to evaluate ecological impacts, long-term performance, and applicability under environmentally relevant conditions. Such studies will focus on optimizing release kinetics and material dosages, quantifying algaecide distribution and transformation products within flocculated systems, evaluating ecological safety toward nontarget organisms, and assessing system performance under mesocosm and field-scale conditions. Integration with complementary toxin mitigation strategies, such as activated carbon or biochar, may further enhance the effectiveness of this approach for HAB management in freshwater systems.

From a feasibility standpoint, the system is compatible with existing PAC based water treatment technologies, which supports its potential for practical implementation. The use of HNT as both a ballast and delivery vehicle further reduces the need for additional materials. We envision field deployment as involving mixing premade loaded and wax-coated HNT with PAC dissolved in water and spraying the slurry over blooms. The deployment will require stockpiles of loaded and wax coated HNT. Careful cost analysis needs to be done before commercial implementation with the possibility that mixtures of HNT and much less expensive clays such as kaolinite might serve the ballast requirement and yet provide sufficient algaecide release.

## Supplementary Material



## Notes

Figure S1: mechanism for hydrogen peroxide generation from propyl gallate; Figure S2: UV–vis spectrum and calibration curve for propyl gallate; Figure S3: effects of low-dosage propyl gallate on cell density; Figure S4: SEM and TEM of HNT; Figure S5: high loading levels of PG in HNT to show PG deposits around the nanotubes (PDF)

## Abbreviations

HAB: harmful algal bloom
MC: *Microcystis aeruginosa*
PG: propyl gallate
PAC: polyaluminum chloride
HNT: halloysite nanotube
PAM: pulse amplitude modulation fluorimetry
Chl-*a*: chlorophyll-*a*
