# Supplementary material for: A Targeted Mitigation Strategy for Freshwater Harmful Algal Blooms through the Release of a Small-Molecule Polyphenolic Algaecide from Clay Nanotubes
Source: Environ Sci Technol. 2026 Jul 16;60(29):20573–84. doi: 10.1021/acs.est.6c03235 (PMC13421970; doi:10.1021/acs.est.6c03235)
Supplement: Supplementary file 1 [file es6c03235_si_001.pdf]

A Targeted Mitigation Strategy for Freshwater Harmful Algal Blooms through the Release of a Small Molecule Polyphenolic Algaecide from Clay Nanotubes

Pedram AziziHariri, [a] *Borui Wang,* [a] *Monica Brady,* [a] *Istiak Hossain,* [a, b] *Nicholas R. Sandoval,* [a] *William Scott,* [c] *Tim I. McLean,* [d] *Vincent J. Lovko,* [c,*] *Vijay T. John* [a,*]

a. Department of Chemical and Biomolecular Engineering, Tulane University, New Orleans, LA 70118.

b. Department of Biochemistry and Microbiology, North South University, Bashundhara, Dhaka-1229, Bangladesh.

c. Phytoplankton Ecology Research Program, Mote Marine Laboratory, Sarasota, FL 33577.

d. Department of Medical Education, The University of Tennessee Health Science Center, Memphis, TN 38163

Supporting Information

Number of pages: 6

Number of figures: 5

Number of tables: 0

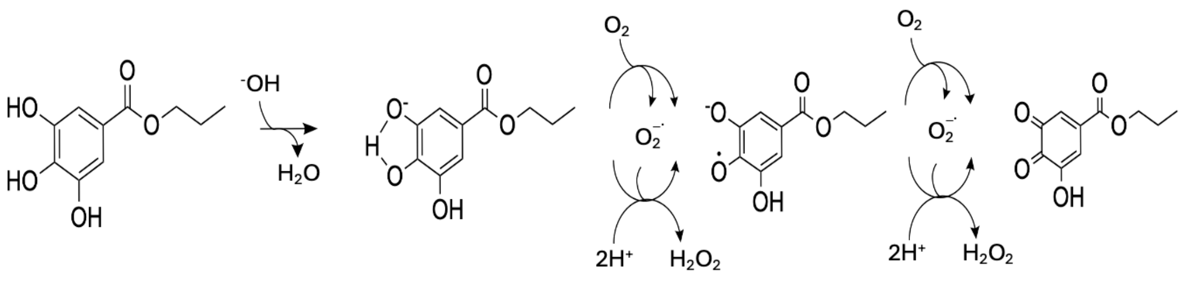

The proposed mechanism for algaecidal activity of propyl gallate through the generation of hydrogen peroxide (adapted from reference 35).

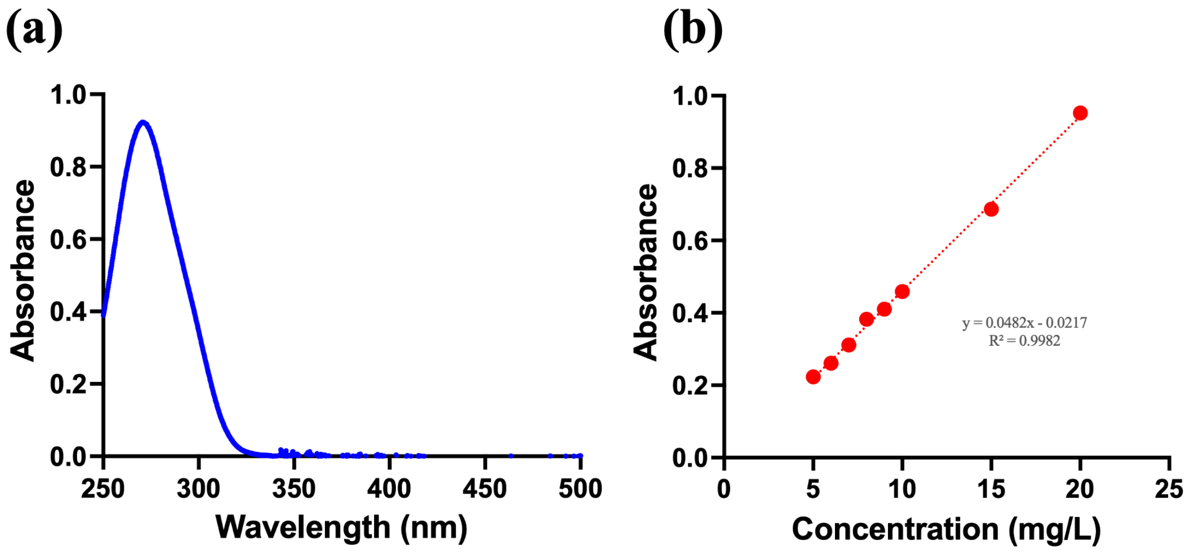

(a) The UV-Vis spectra of propyl gallate around the characteristic peak (~275 nm); (b) the calibration curve of propyl gallate dissolved in DI water.

(a) (b)

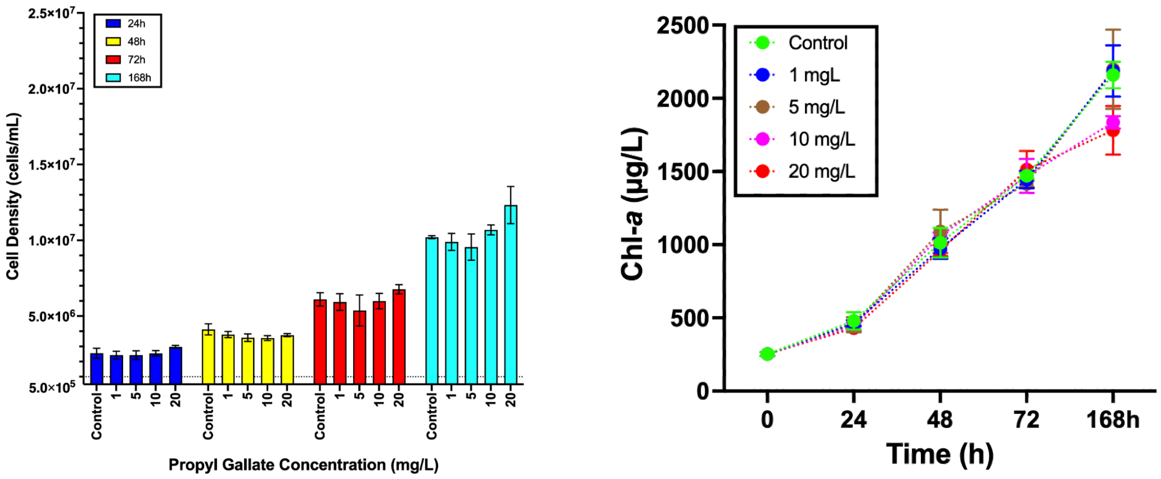

(a) Effect of propyl gallate on the cell density of MC at different concentrations (1, 5, 10, 20 mg/L). Concentrations below 25 mg/L are shown not to have a growth suppression effect within the first seven days; (b) extracted Chl-*a* from MC shows that a concentration up to 20 mg/L PG does not slow down the production of Chl-*a* throughout the seven-day period, while higher concentrations (25 mg/L and onwards), halt the production of Chl-*a*.

(a) **(b)**

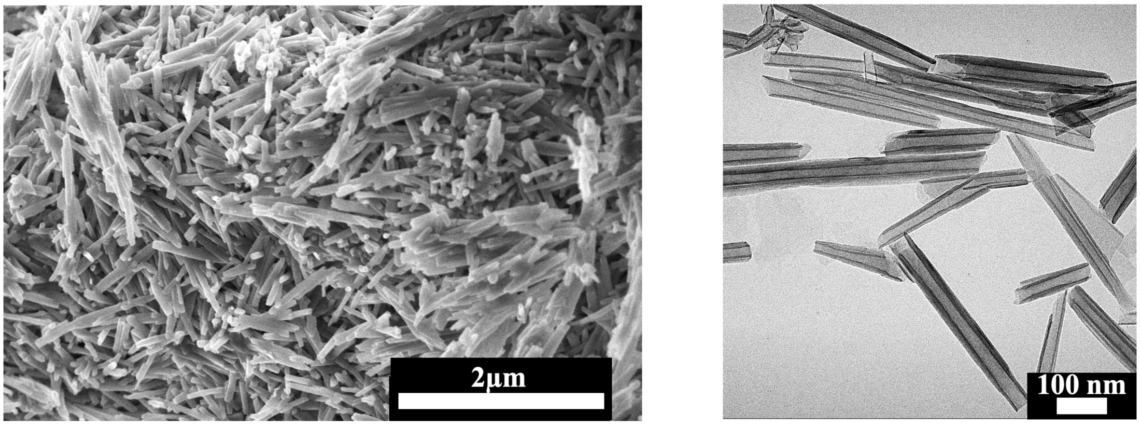

Electron micrographs of HNT. (a) an SEM micrograph of HNT, showing their rod-like morphology; (b) a TEM micrograph, illustrating the empty lumens of HNT.

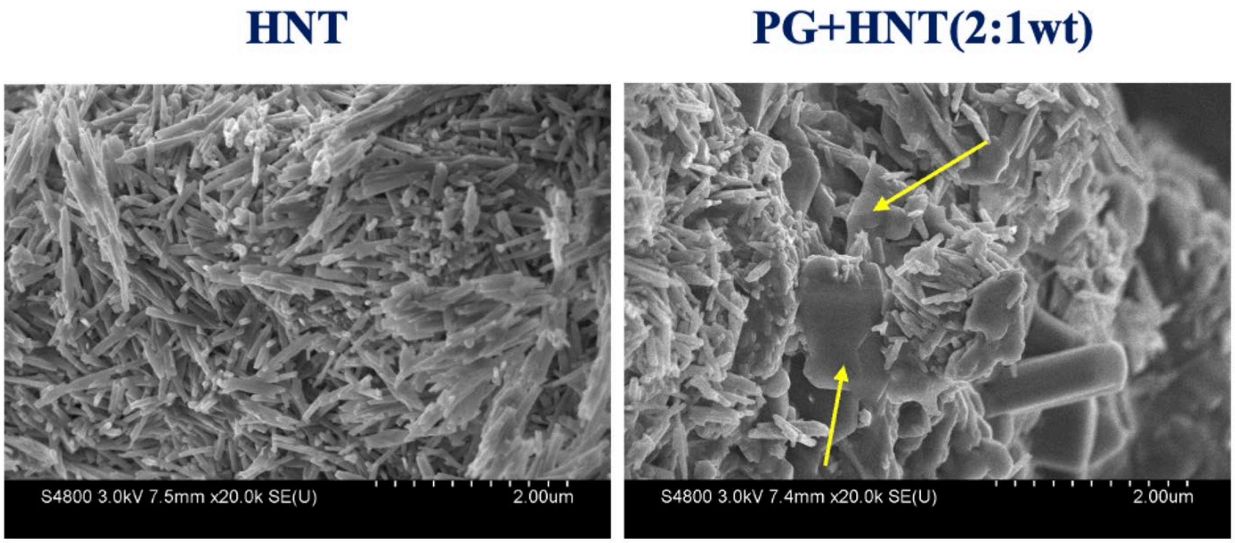

SEM images of HNT loaded with PG. PG was mixed with HNT at 2:1 weight ratio in methanol. Methanol was then evaporated in a rotary evaporator, and the remaining solid was ground prior to SEM. The deposits around the nanotubes evidence the presence of propyl gallate.
